# Low-Cost ZnO Spray-Coated Optical Fiber Sensor for Detecting VOC Biomarkers of Diabetes

**DOI:** 10.3390/s23187916

**Published:** 2023-09-15

**Authors:** Kankan Swargiary, Pannathorn Jitpratak, Akhilesh Kumar Pathak, Charusluk Viphavakit

**Affiliations:** 1 International School of Engineering (ISE), Intelligent Control Automation of Process Systems Research Unit, Chulalongkorn University, Bangkok 10330, Thailand; 2 Biomedical Engineering Program, Faculty of Engineering, Chulalongkorn University, Bangkok 10330, Thailand; 3 Center for Smart Structures and Materials, Department of Mechanical Engineering, Northwestern University, Evanston, IL 60208, USA

**Keywords:** optical fiber sensor, volatile organic compound (VOC), ZnO spray coating, biomarkers, diabetes

## Abstract

A non-invasive optical fiber sensor for detecting volatile organic compounds (VOCs) as biomarkers of diabetes is proposed and experimentally demonstrated. It offers a low-cost and straightforward fabrication approach by implementing a one-step spray coating of a ZnO colloidal solution on a glass optical fiber. The structure of the optical fiber sensor is based on a single-mode fiber–coreless silica fiber–single-mode fiber (SMF-CSF-SMF) structure, where the CSF is the sensor region spliced between two SMFs. The ZnO layer of a higher refractive index coated over the sensing region improves the light interaction with the surrounding medium, leading to sensitivity enhancement. The optical properties, morphology, and elemental composition of the ZnO layer were analyzed. The sensing mechanism of the developed sensor is based on a wavelength interrogation technique showing wavelength shifts when the sensor is exposed to various VOC vapor concentration levels. Various concentrations of the three VOCs (including acetone, isopropanol, and ethanol) ranging from 20% to 100% were tested and analyzed. The sensor noticeably shows a significant response towards acetone vapor, with a better sensitivity of 0.162 nm/% vapor than for isopropanol (0.082 nm/% vapor) and ethanol (0.075 nm/% vapor) vapors. The high sensitivity and selectivity towards acetone, a common biomarker for diabetes, offers the potential for further development of this sensor as a smart healthcare system for monitoring diabetic conditions.

## 1. Introduction

Diagnosing various health-related diseases by exhaled breath analysis has attracted many researchers and is considered to be of strong interest [1]. Various odors from exhaled breath have already been detected by many physicians during classical times that are related to specific health diseases, such as a ‘fruity’ odor from diabetic patients [2]. These odors are the volatile organic compounds (VOCs) recognized as disease biomarkers in exhaled breath [3]. Exhaled breath contains thousands of different VOCs generated from the body’s metabolic process. Some VOCs in the exhaled breath of diabetic patients [1] are higher than in healthy people due to chronic metabolic disorders [4]. The human body is designed to store a constant glucose level in the blood from the food intake for energy with the help of the insulin hormone. Glucose levels in the blood of those with diabetes are higher than usual due to inadequate insulin or cells that are unresponsive to insulin. Therefore, regular blood glucose monitoring is necessary to track diabetes management to prevent complications, such as heart attack, stroke, kidney diseases, nerve damage, and vision loss [5]. The most common method to monitor blood glucose levels involves using blood tests, which is an invasive method. It can be time-consuming, intrusive, and painful [6]. Therefore, a non-invasive method to monitor the condition of diabetes has been proposed. People with diabetes burn fats instead of glucose, producing massive amounts of ketones (such as acetone), which are then released from the respiratory tract. VOC biomarkers from the exhaled breath of diabetic patients have been studied in the health industry for screening and diagnosis due to the non-invasiveness and cost-effectiveness of the approach. This painless technique offers rapid qualitative and quantitative detection [7]. People with different types of diabetes display several VOCs in their exhaled breath, such as acetone, isopropanol, and ethanol [8]. Acetone is considered the most common biomarker for type-1 diabetes and type-2 diabetes. Its concentration is directly related to blood glucose levels [9,10,11].

Acetone is mainly produced by the decarboxylation of acetoacetate derived from lipolysis, known as beta-oxidation. In healthy individuals, acetone presents in a low amount of 0.8 parts per million (ppm), which is considered a normal breath constituent. In diabetic patients, the acetone concentration in exhaled breath exceeds 1.76 ppm [12,13]. In addition, the study by Chuang et al. reported that if the acetone concentration in exhaled breath is more than 1.71 ppm, a person has type-2 diabetes, and a person with more than 2.19 ppm shows type-1 diabetes [14]. However, the acetone concentration in the human breath can be increased during fasting, exercise, or a ketogenetic diet [15]. Therefore, analyzing the acetone levels from exhaled breath can be an alternative method for diagnosing and monitoring patients’ diabetic conditions.

Currently, acetone levels can be accurately detected by Gas Chromatography–Mass Spectrometry (GC–MS), Selected Ion Flow Tube Mass Spectrometry (SIFT-MS), Proton Transfer Reaction Mass Spectrometry (PTR-MS), Laser Photoacoustic Spectrometry (LPS), and Gas Chromatography–Mass Spectrometry with Solid-Phase Microextraction (SPME-GC-MS) [10,16,17,18,19,20,21]. These techniques are reliable, sensitive, and have a remarkable potential for breath analysis to measure breath. However, these methods are time-consuming, expensive, and unsuitable for routine diabetic monitoring. They require highly skilled people to operate their sophisticated systems. Therefore, the recent development of low-cost, efficient, and high-sensitivity sensors that can operate at room temperature (RT) with different humidity levels is necessary. Many researchers have reported a new development of VOC biomarker sensors, such as electrochemical [22,23], resistive [24,25], and optical sensors [26]. However, resistive and electrochemical sensors have limitations on selectivity and shelf life. They are also affected by humidity. On the other hand, optical sensors receive high attention in chemical, gas, and biosensing due to their robust nature, small size, high sensitivity, remote sensing capability, immunity to electrical noises, multiplexing, and real-time monitoring [27,28,29]. Optical fiber-based VOC sensors have found significant application in industrial sectors, revolutionizing air quality monitoring and process control. These sensors utilize the principles of light absorption and reflection to detect and quantify VOC concentrations, enabling real-time and accurate measurement. Their non-invasive nature allows for remote monitoring of hazardous environments, reducing the need for human intervention and ensuring worker safety [29]. An optical fiber sensor with a single-mode–multimode–single-mode (SMS) structure has been reported for VOC biomarker detection by coating novel materials in the sensing part of the fiber [26,30,31]. Currently, several materials are being employed for VOC detection, such as graphene, conducting polymers, and metal oxides, such as zinc oxide (ZnO), titanium dioxide (TiO_2_), tin oxide (SnO_2_), and nickel oxide (NiO). Among them, ZnO offers and shows the potential to improve overall sensitivity and selectivity performance due to the capability of detecting VOCs at ppb, ppm, and ppt levels with a fast response and recovery time, unlike conducting polymers, graphene, and other metal oxide families, which lack selectivity and response-time performance [26,32,33,34].

ZnO is one of the most promising candidates for VOC sensing. It has a wide band gap of 3.37 eV at 300 K and a large excitation binding energy of 60 meV at RT. ZnO also has vast applications in the industrial sectors, such as electronics, cosmetics, medical, food, automobile, and textiles industries, due to its high surface-to-volume ratio, high stability, unique electrical and optical properties, high chemical sensitivity, good transparency, and high biocompatibility. Due to the unique ZnO characteristics, it is also found to be implemented to develop high-performance VOC sensors with cost-effectiveness [35]. ZnO nanostructures of different morphologies coated over optical fiber sensors are reported to have high sensitivity, selectivity, and improvement in response time, as well as the possibility of mass production. Various nanostructures of ZnO, including nanoparticles, nanowires, nanoflakes, nanorods, nanotubes, and thin films, have been reported in the application of VOC vapor sensing to show different sensitivities towards different VOCs. ZnO-coated sensors have also been reported to be unaffected by humidity, which is very beneficial in breath analysis, as the humidity in exhaled breath is excessive [36].

The present work focuses on developing ZnO nanoparticles coated on a single-mode fiber–coreless silica fiber–single-mode fiber (SMF-CSF-SMF)/SCS sensor to detect VOC biomarkers of diabetes. The sensor was tested using different concentrations of three VOC vapors, including acetone, ethanol, and isopropanol, from 20% to 100%. The length of the CSF region was optimized by theoretical modeling to attain the maximum sensitivity of the system. The sensing performance was executed to study the sensitivity, selectivity, and repeatability of the device. Our proposed fabricated sensor offers a simple and cost-effective fabrication technique ideal for integration into smart healthcare systems.

## 2. Materials and Methods

### 2.1. Materials and Reagents

All the chemicals and reagents were of analytical grade and used without further purification. Zinc acetate 2-hydrate (Zn (CH_3_COO)_2_·2H_2_O) (KemAus, Cherrybrook, NSW, Australia) and potassium hydroxide (KOH) pellets (Merck KGaA, Sigma Aldrich, Burghausen, Germany) were used as precursors. Ethanol (C_2_H_6_O) (QReC, Auckland, New Zealand) was used as a solvent. An SMF 28 with a core/cladding diameter of 8.2 μm/125 μm (Thorlabs, Newton, NJ, USA) and a CSF with a diameter of 125 μm (YOFC, Wuhan, China) were used to construct the SMF–CSF–SMF structure.

### 2.2. Sensor Design

**Figure 1:**
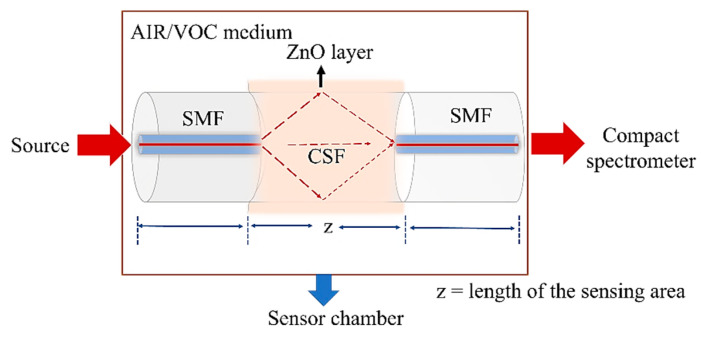
Schematic diagram of the ZnO-coated optical fiber sensor structure.

The sensor is proposed to have a single-mode fiber–coreless silica fiber–single-mode fiber (SMF–CSF–SMF) structure composed of a short section of the CSF spliced between the two SMFs, as shown in Figure 1. The CSF region is the sensing area, where more modes can propagate inside, creating multimode interference (MMI). This sensor works based on the evanescent field sensing mechanism. The change in MMI due to the change in the refractive index of the surroundings is detected by the evanescent fields at the sensor interface. This MMI increases the intensity of the evanescent field at the CSF interface compared to the SMF and conventional MMF, offering higher interaction with the surroundings. A ZnO layer with a high refractive index of n = 1.7–2.5 [37] is coated over this CSF area, offering a large surface area in the sensing region. The light is guided into the ZnO nanostructure because of its higher refractive index along with its morphology and the thickness of the coating layer, causing a larger volume of light interaction at the interface. This provides an enhancement in the light and surrounding medium interaction at the evanescent region of the fiber interface at the evanescent region of the fiber interface, which plays a significant role in the performance of the sensor. With the presence of VOCs in the surrounding medium, the modal property and the propagation constants of the light guided in the sensing region change due to variation in the refractive index, which can be detected from the wavelength shift in the absorption spectrum.

MMI in the sensing region is changed due to the variation in the refractive index of the surrounding medium. The detailed theory is well established and has been explained in previous reports [6,26,38,39,40]. At any distance in the CSF, the electric field ($E_{CSF}$) is given as:

(1) $$E_{CSF}\left(r,z\right)=\overset{N}{\underset{n=1}{\sum }}a_{n}\psi_{n}\left(r\right)\exp\left(-j\beta_{n}z\right)$$

where $a_{n}$ is the amplitude coefficient for each guided radial mode with the profile $\psi_{n}\left(r\right)$. When each mode propagates, a different phase is gained based on its propagation constant. $\beta_{n}$ is the longitudinal propagation constant of the *n*th guided radial mode.

At the output, the total coupling or coupling efficiency (η) in the fiber is calculated from the output intensity, which is from the light at the end of the CSF region, and the input intensity, which is the light inside the SMF region, as shown in the Equation (2),

(2) $$\eta =\frac{{\left|\int_{r=0}^{\infty }E_{CSF}\left(r,z\right)E_{s}\left(r\right)dr\right|}^{2}}{\int_{r=0}^{\infty }I_{CSF}\left(r,z\right)dr\int_{r=0}^{\infty }{\left|E_{s}\left(r\right)\right|}^{2}dr}$$

**Figure 2:**
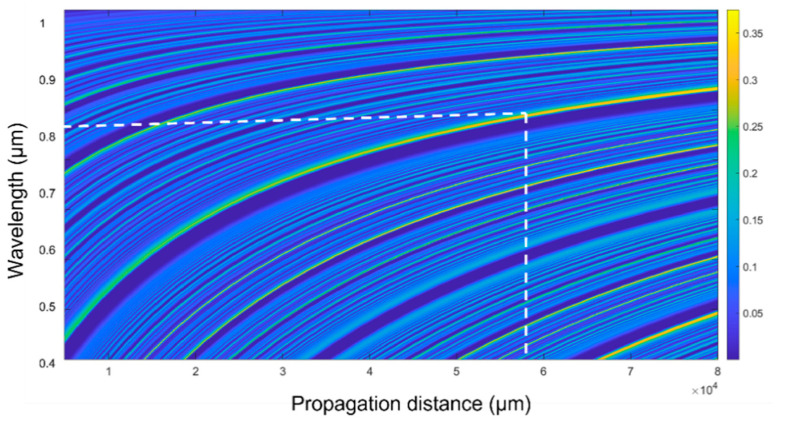
Coupling efficiency variation at different propagation distances in the CSF region over a broad wavelength.

The coupling efficiency is calculated to obtain the propagation distance in the sensing region that provides maximum intensity at the output. The distance is called the re-imaging distance, as it has a similar optical field profile to the input. With the maximum intensity at the output, the light interaction volume with the surrounding medium increases, leading to high sensitivity achievement. Therefore, the re-imaging distance is considered the length of our sensor to achieve the maximum possible sensitivity. The coupling efficiency at different distances in the sensing region (z) over a broad wavelength (100–1000) nm is shown in Figure 2. At the wavelength around 820 nm, the re-imaging distance, where the maximum coupling efficiency is obtained, is 5.8 cm for a 0.07 volume fraction of ZnO at the interface. Therefore, the sensor is designed to have a sensing region of 5.8 cm to achieve the maximum possible output signal.

### 2.3. Synthesis of Colloidal ZnO Solutions

**Figure 3:**
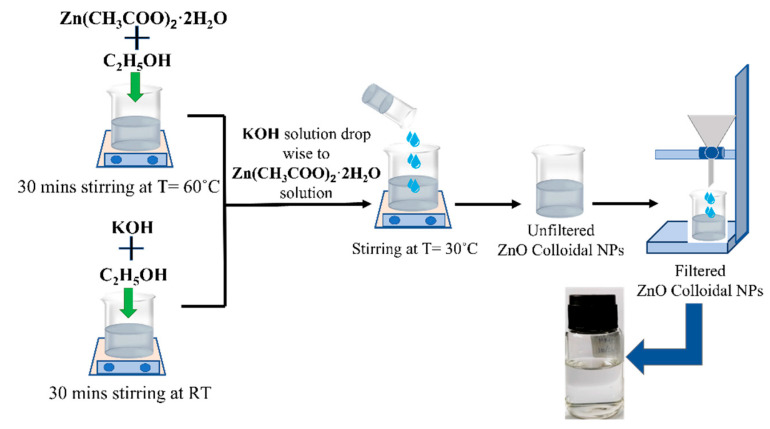
ZnO colloidal nanoparticle synthesis process.

The ZnO colloidal nanoparticles (NPs) were synthesized by the aqueous chemical method. Zn(CH_3_COO)_2_·2H_2_O and KOH are precursors with ethanol as a solvent in the synthesis [41,42,43]. Firstly, 0.1 M of Zn(CH_3_COO)_2_·2H_2_O is dissolved in 15 mL of ethanol with vigorous stirring at 60 °C. At the same time, 0.1 M of 15 ml KOH solution with ethanol is also prepared in a separate beaker at RT. These mixtures are stirred continuously for 30 min until a homogenous mixture of zinc salt and complete dissolution of KOH is obtained. Then, the KOH solution is added dropwise to the Zn(CH_3_COO)_2_·2H_2_O solution after cooling to 30 °C under continuous stirring (600 rpm). The solution is finally filtered with 2.5 µm filter paper to obtain a clear and colorless solution with pH~7. The schematic diagram of the ZnO colloidal NP synthesis is shown in Figure 3.

### 2.4. Sensor Fabrication

**Figure 4:**
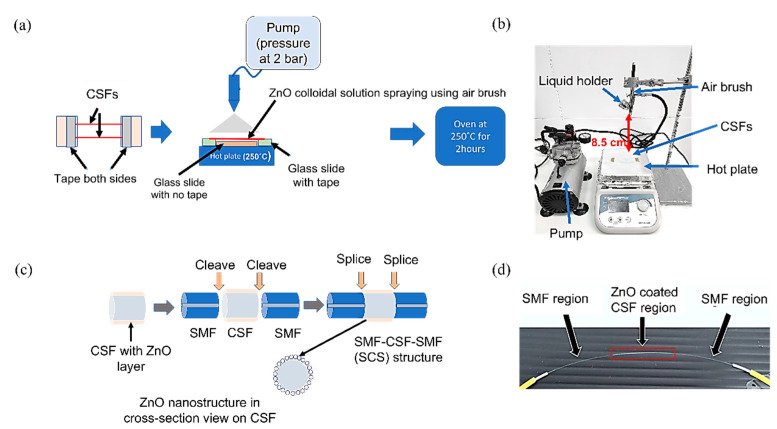
(**a**) Schematic diagram of the spray-coating process. (**b**) Actual image of the spray-coating setup. (**c**) Schematic diagram of the fiber splicing process. (**d**) Image of the fabricated sensor.

A 5.8 cm long CSF was spray-coated with a layer of ZnO NPs first before connecting with two SMFs to obtain an SMF-CSF-SMF (SCS) configuration. The fabrication steps for the spray coating of the clear ZnO solution onto the CSFs are shown in Figure 4a,b. A cost-effective airbrush is used in the spray coating process. The CSFs are cleaned and pre-heated on a hot plate at 250 °C for 10 s. Then, 0.2 mL of ZnO solution is sprayed on the pre-heated CSFs with a pressure of 2 bar for 15 s. The distance between the CSFs and the airbrush is set at 8.5 cm. The fiber is rotated while spraying to obtain uniformity of the ZnO layer. Then, the fiber is post-heated on the hot plate for 10 s to ensure that the nucleation process occurs. After that, the CSFs coated with the ZnO layer are further annealed in an oven at 250 °C for 2 h. A uniform distribution of ZnO nanoparticles on the optical fiber is obtained by spraying a homogeneous ZnO colloidal solution while rotating the fiber. A uniform ZnO layer provides a larger surface area for interaction between the nanoparticles and VOCs. In addition, a uniform layer offers a uniform surface property to the sensor, leading to good sensor consistency. It is worth mentioning that the desired uniformity and thickness of the ZnO layer is achieved after repeating the optimized fabrication parameters more than 10 times for each different fiber. In the next step, the ZnO-coated CSFs are spliced with two SMFs at both ends by using a fiber splicer (Sumitomo Electric, Z2C, Osaka, Japan) to obtain a configuration of the SMF-CSF-SMF (SCS) structure. In order to ensure a good connection between the CSF and the SMF, the splicing loss has to be less than 0.01 dB after the splicing process. A schematic diagram of the fabrication steps of the SCS structure, along with the ZnO nanostructure, is shown in Figure 4c, and the actual image of the fabricated SCS structure is shown in Figure 4d.

### 2.5. Optical Setup

**Figure 5:**
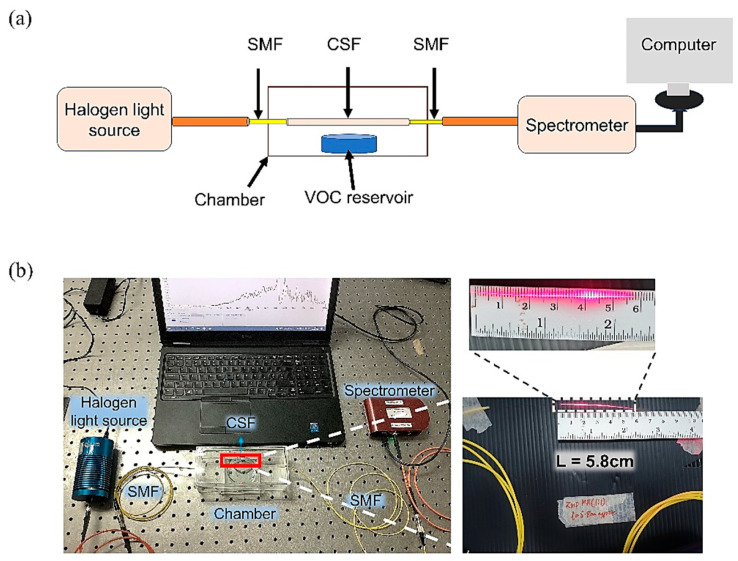
(**a**) Schematic diagram of the optical setup. (**b**) Actual image of the optical setup. Inset shows the fabricated CSF sensor after spray coating the ZnO layer. The sensor is 5.3 cm long.

In this work, the fabricated sensor was tested with 3 different VOC vapors: acetone, ethanol, and isopropanol, as they are common biomarkers for diabetes [44]. The sensor works based on the wavelength interrogation in which the wavelength shift is observed in the output spectrum when there is a change in the refractive index of the surrounding medium. The optical setup includes a halogen light source (Ocean Optics, HL-2000, Largo, FL, USA) connected to the fabricated sensor, which is placed inside a gas chamber. At the other end of the sensor, it is connected to a CCD spectrometer (Thorlabs, CCS200/M, Newton, NJ, USA) with an operating wavelength range of 200–1000 nm to observe the output spectrum and its shift. A schematic of the optical setup is shown in Figure 5a, and the actual setup is shown in Figure 5b.

### 2.6. VOC Vapor Preparation

The experiment involved preparing acetone, ethanol, and isopropanol vapors by placing their solutions in a VOC reservoir inside a chamber to naturally evaporate at room temperature (RT). These vapors interacted with a ZnO nanostructure layer coated on the sensor, causing a shift in the absorption spectrum due to changes in the refractive index of the surroundings. To establish a baseline, the ZnO-coated sensor was first exposed to DI water, representing 0% of VOC vapor, and its output spectrum was recorded as a reference ($I_{0})$. After that, the sensor was exposed to each VOC vapor at different concentrations of 20%, 40%, 60%, 80%, and 100%. These concentrations were achieved by vaporizing the VOC solutions in DI water. The spectrometer recorded the optical spectra for 15 min after sealing the chamber. The entire experiment was conducted at RT. To ensure the consistency of the sensor, the experiment was conducted 3 times to evaluate both the repeatability and stability of the fabricated fiber sensor. Any absorbed VOCs in the sensor and chamber were removed using nitrogen (N_2_) gas before each new measurement.

## 3. Results and Discussions

### 3.1. Optical Properties of the Prepared ZnO Colloidal Solution

**Figure 6:**
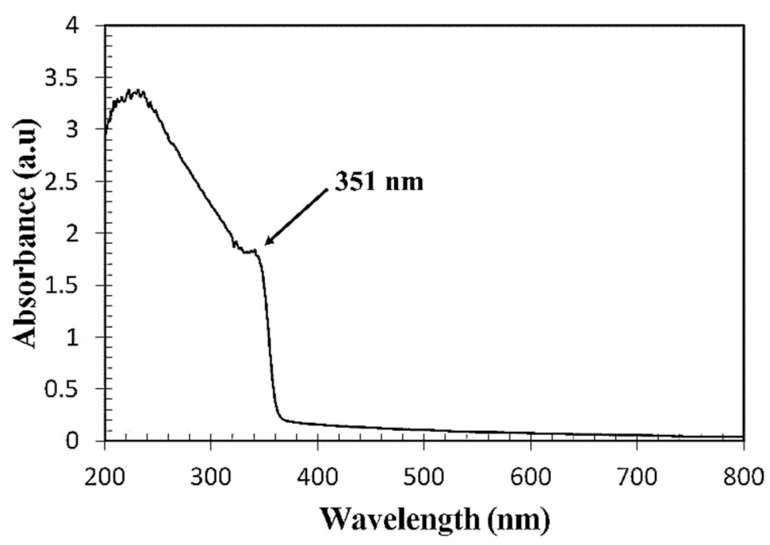
UV-Vis absorption spectra of the synthesized ZnO colloidal solution.

The optical properties of the ZnO colloidal solution were investigated from its absorption spectrum using a UV-Vis spectrophotometer (PerkinElmer, LAMBDA 850+, Waltham, MA, USA) in the 200–800 nm wavelength range, as shown in Figure 6. The absorption peak observed at 351 nm indicates the intrinsic bandgap of Zn-O absorption. It displays a monodispersed nature of the particle size distribution, which confirms the formation of ZnO NPs.

### 3.2. Particle Size Distribution and Surface Charges of the ZnO Colloidal Nanoparticles

**Figure 7:**
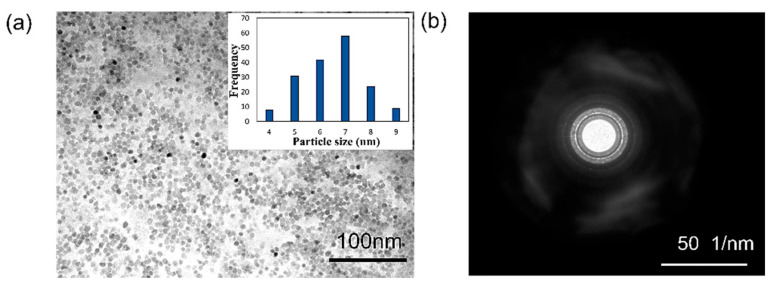
(**a**) TEM of the synthesized ZnO nanoparticles. Inset shows the particle size distribution. (**b**) The SAED pattern.

The size distribution of the ZnO particles affects the sensitivity of the sensor. With the same volume of the ZnO layer, the smaller the particle size, the larger the surface area. This improves the sensitivity due to a larger amount of light interaction with the targeted VOC. A zeta sizer (MALVERN, Nano ZSP, Malvern, UK) was used to analyze the particle size distribution of the clear ZnO colloidal solution through dynamic light scattering (DLS). The average ZnO particle size of 10 nm was well-dispersed and uniform. Further confirmation of the particle size and shape was obtained using a transmission electron microscope (TEM; JOEL, JEM-2100, Akishima, Japan). The TEM image showed ZnO NPs with a mean size of 7 nm and a narrow size distribution, as shown in Figure 7a. The selected area electron diffraction (SAED) pattern revealed distinct rings, confirming the nano-polycrystalline nature of the ZnO NPs, as shown in Figure 7b.

Zeta potential analysis was also carried out to check the stability of the ZnO NPs and their surface charges. The zeta potential of the ZnO was around 7.2, indicating that the surface charge was neutral. This confirms that the ZnO solution was stable. However, agglomeration of the ZnO solution was possible after 24 h, and the remaining solution was discarded.

### 3.3. Surface Morphology of the ZnO Layer Coated on the Fiber Sensor

**Figure 8:**
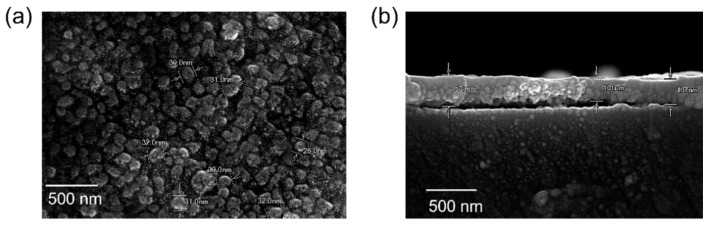
FE-SEM images of the ZnO-coated fiber sensor. (**a**) Top surface of the ZnO-coated fiber sensor. (**b**) Cross-sectional image showing the thickness of 103 nm.

The morphological analysis of the ZnO-coated fiber sensor was conducted using a field emission scanning electron microscope (FE-SEM; Hitachi, FESEM, SU-8230, Tokyo, Japan). A top view and cross-sectional images of the ZnO layer coated on the fiber sensor are shown in Figure 8a,b, respectively. It can be observed that the ZnO layer is 103 nm thick and that the ZnO nanoparticles have an average size of 30 nm. This is slightly higher than the average size of the colloidal nanoparticles due to agglomeration during the spray coating process. However, the ZnO layer is uniformly deposited over the CSF surface, ensuring a great sensing performance and efficiency. The small size of the ZnO nanoparticles and their uniform distribution provide a large surface area, enhancing the interaction for molecular absorption of VOC vapors. This contributes to the sensitivity of the sensor and effectiveness in detecting VOCs.

### 3.4. Saturation Time of VOC Absorption on the Fiber Sensor

**Figure 9:**
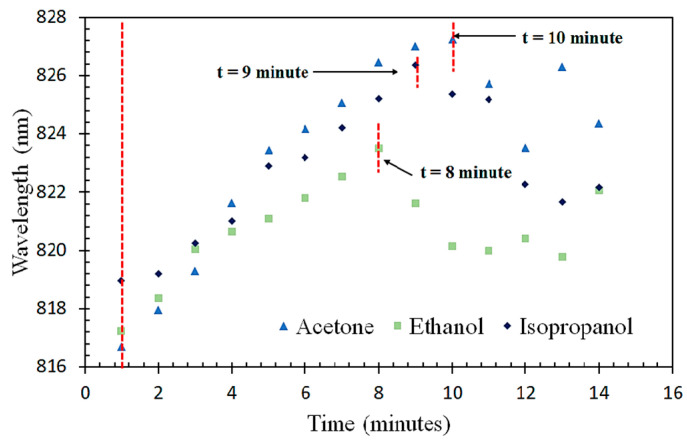
Saturation times of the ZnO-coated fiber sensor for VOC vapors.

The kinetic absorption of VOC vapors onto the sensor was studied to investigate the saturation time, where the maximum sensitivity could be achieved for all the VOC vapors (acetone, ethanol, and isopropanol). The sensor was exposed to a 100% VOC concentration vapor continuously for 15 min. The peak wavelength shifts over time were observed and recorded (Figure 9). The results show that, for all three VOC vapors, the sensor experienced a redshift in wavelength over time. The saturation times for the ZnO-coated optical fiber sensor to fully absorb the acetone, isopropanol, and ethanol vapors were found to be 10 min, 9 min, and 8 min, respectively. This information provides valuable insights into the response time of the sensor for each VOC, aiding in its practical application for gas detection.

The absorption characteristics of the VOC vapors on the sensor can be varied at different concentrations at a fixed time [26]. Lower concentrations of VOC vapors (20%, 40%, 60%, and 80%) lead to varying absorption on the ZnO layer due to a reduced evaporation volume, resulting in possible wavelength shifts. Based on this principle, further investigations were carried out by exposing the sensor to lower concentrations, using the individual saturation times obtained for each of the three VOCs.

### 3.5. Sensing Performance

**Figure 10:**
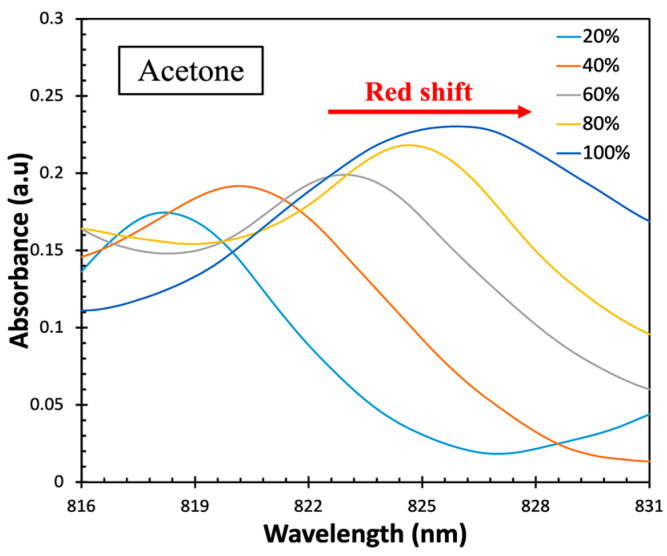
Absorption spectra of the ZnO-coated CSF sensor with different concentrations of acetone vapor.

The different concentrations of each VOC were prepared to investigate the sensor response to the three VOCs, as mentioned in Section 2.6. First, the sensor was exposed to DI water (0% VOC vapor) as a reference to minimize the effect of the water evaporation. Then, the sensor was exposed to various concentrations (20%, 40%, 60%, and 80%) of acetone vapor. The measurements were carried out for 10 min based on the kinetic absorption of acetone on the surface of the sensor. The experiment was repeated three times to ensure the reliability and consistency of the sensor. The same procedure was followed on the same sensor for isopropanol and ethanol at RT. The collected data were analyzed using the wavelength interrogation technique, which was the primary sensing mechanism used in this work. The absorption spectra (A) of acetone vapor, as shown in Figure 10, were obtained from Equation (3).

(3) $$A=-\log\frac{I}{I_{o}}$$

where $I_{o}$ is the reference intensity from the sensor exposed to DI water (0% VOC vapor) and I is the transmitted intensity obtained when the sensor is exposed to different VOC vapors at different concentrations from 20% to 100%.

**Figure 11:**
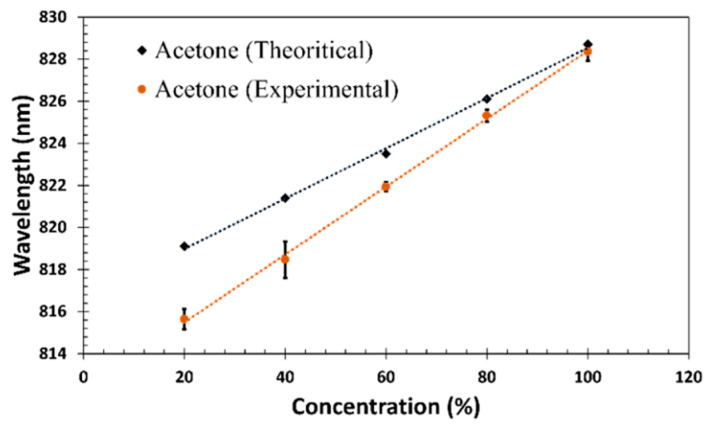
Comparison of the experimental and theoretical results of peak wavelength with acetone vapors.

The results showed wavelength shifts at λ = 816–828 nm in the absorption spectra when the ZnO-coated fiber sensor was exposed to various concentrations of acetone vapor at a fixed time. These shifts occurred due to changes in the refractive index at the ZnO layer during VOC absorption. As the concentration of acetone vapor increased, a redshift in the spectra was observed. The peak wavelength for each acetone concentration absorption closely aligned with the theoretical simulation, as shown in Figure 11.

**Figure 12:**
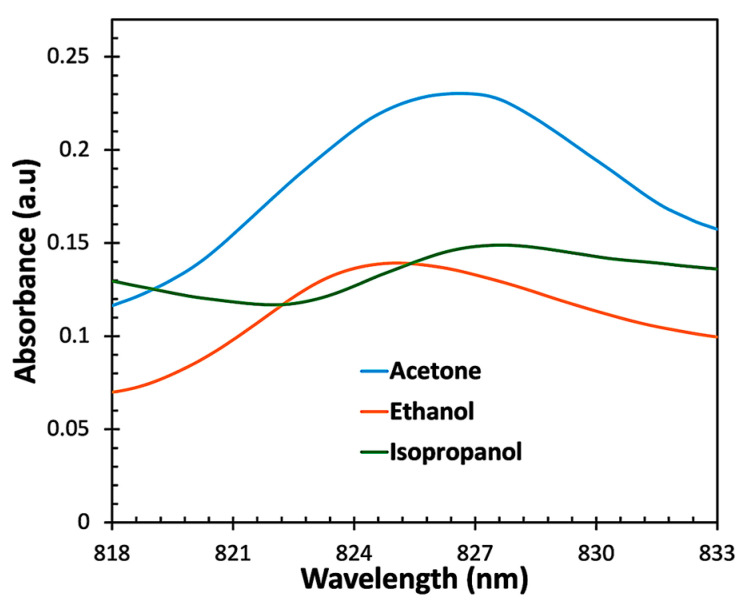
Absorbance of the ZnO-coated optical fiber sensor for acetone, ethanol, and isopropanol vapors.

This good agreement between the experimental and theoretical results confirms the accuracy and reliability of the sensor in detecting acetone vapor across various concentration levels. From this validation, the sensor was then further evaluated with ethanol and isopropanol to investigate the absorption selectivity of the fabricated sensor to different VOCs, as shown in Figure 12.

**Figure 13:**
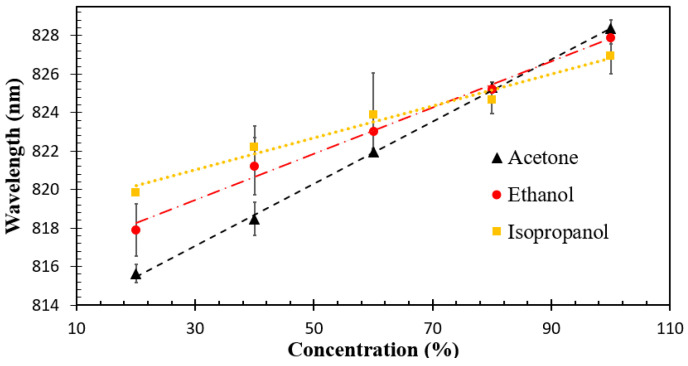
Sensitivity of the ZnO-coated optical fiber sensor for acetone, ethanol, and isopropanol vapors.

From Figure 12, it can be seen that the sensor exhibited the highest absorbance to acetone vapor compared to ethanol and isopropanol vapors. This can be attributed to the spherical structure of the ZnO nanoparticles, which provides an active site on the surface of the sensor, facilitating the reaction between light and acetone molecules. Consequently, acetone molecules react selectively at the sensing region, leading to higher sensitivity and selectivity than other VOC molecules. To obtain the sensitivity of the sensor, the peak wavelengths in the absorption spectra of ethanol and isopropanol were measured and compared to those of acetone, as shown in Figure 13.

Figure 13 shows that redshifts occurred when the sensor was exposed to higher concentrations of acetone, ethanol, and isopropanol vapors. The graph exhibits a strong correlation between VOC vapor concentration and peak wavelength, with regression values of 0.99, 0.98, and 0.97 for acetone, ethanol, and isopropanol, respectively. This excellent regression allowed us to calculate the linear sensitivity of the sensor using Equation (4).

(4) $$Sensitivity\;\left(nm/\%VOC\;vapor\right)=\frac{\Delta Wavelength}{\Delta Concentration\;of\;VOC\;vapor}$$

where ΔWavelength is the change in the peak wavelength and ΔConcentration of VOC vapor is the change in the VOC concentration.

The sensitivity values of the sensor were found to be 0.16 nm/% for acetone vapor, 0.08 nm/% for IPA vapor, and 0.07 nm/% for ethanol vapor. These results indicate that the ZnO-coated optical fiber sensor exhibits higher sensitivity to acetone vapor, with its morphology playing a vital role in enhancing sensing performance. When the ZnO interacts with alcohol vapor, it induces changes in the effective refractive index at the outer cladding, resulting in significant improvements in the light interaction within the evanescent field region.

The performances of our fabricated sensor and the other optical sensors from the available literature are listed below in Table 1.

The proposed sensor exhibited a significant response in terms of sensitivity and selectivity to acetone vapor among the other tested VOCs. To the best of our knowledge, the structure of our proposed sensor has been utilized for the first time in sensing VOCs in the vapor form, along with the selectivity tests. The performance measurements indicate the potential for integration into a larger healthcare system or network, particularly in detecting VOC biomarkers from the exhaled breath of diabetic patients. It is worth mentioning that in the case of mixed VOC biomarkers, further evaluation will be conducted in the future by incorporating a bundle of fibers with different ZnO morphological nanostructures and combing it with different materials to selectively detect each individual biomarker.

## 4. Conclusions

A low-cost optical fiber sensor with a ZnO nanostructure can be achieved through one-step spray coating, which is a simple and cost-effective process. This sensor is designed for VOC biomarker sensing, especially for diabetes. The sensor structure consists of a uniform ZnO nanoparticle film spray-coated on a coreless silica fiber (CSF) sensor region with a length of 5.8 cm integrated between two single-mode fibers (SMFs) to create an SMF-CSF-SMF optical device. Utilizing the wavelength interrogation technique, our proposed sensor exhibits a sensitivity of 0.162 nm/% vapor and selectivity to acetone, the most common VOC biomarker for diabetes. The sensor offers a low-cost approach with a non-invasive nature, and it holds the potential for integration into a sensor network to monitor and estimate glucose levels through VOC biomarkers in the exhaled breath of diabetic patients. With its promising performance, the ZnO-coated optical fiber sensor provides new possibilities for improved diabetes care and smart healthcare solutions.

## Figures and Tables

**Table 1:** Comparison of the various optical fiber sensors in sensing different VOCs.

**Material and Method**	**Structure**	**Test Gases**	**Concentration**	**Sensitivity/Response**	**Response Time**	**Working Temperature**	**Ref.**
ZnO nanorods (Hydrothermal)	SMS fiber structure	Isopropanol	20–100%	0.053 nm/% IPA vapor	9 min	RT	[26]
ZnO nanoparticles (Aqueous chemical route)	Clad-modified optical fiber	Acetone	50–250 ppm	14	NA	RT	[45]
ZnO film (Atomic layer deposition)	SMS fiber structure	Ethanol	50–100%	50% ethanol:0.065 fitted curve 62% ethanol:0.056 fitted curve	5 min	RT	[46]
Nanocrystalline ZnO	Clad-modified optical fiber	Acetone	0–500 ppm	−0.27 counts/100 ppm	48 min	RT	[47]
Magnesium cobalt oxide (MgCo_2_O_4_) (Hydrothermal)	Clad-modified optical fiber	Acetone	500 ppm	42 × 10^−3^ k/Pa	25 s	RT	[48]
ZnO nanoparticles (Aqueous chemical route)	SMS fiber structure	Acetone, ethanol, and isopropanol	20–100%	0.16 nm/% acetone vapor 0.08 nm/% IPA vapor 0.07 nm/% ethanol vapor	Acetone: 10 min Isopropanol: 9 min Ethanol: 8 min	RT	Present work

## Data Availability

Not applicable.
